# Photo Quiz

**DOI:** 10.3201/eid1409.086671

**Published:** 2008-09

**Keywords:** pathology, cell pathology, pathologic anatomy, photo quiz

**Figure:**
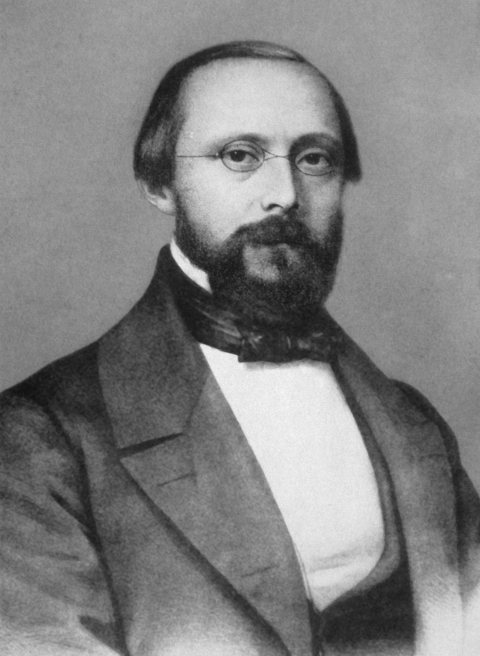


## Who is this man and what did he accomplish?

**Here is a clue. He said:**

“For if medicine is really to accomplish its great task, it must intervene in political and social life. It must point out the hindrances that impede the normal social functioning of vital processes, and effect their removal.”

**Is he:**

A) Robert Koch
B) Charles Nicolle
C) Louis Pasteur
D) Rudolf Virchow
E) Max von Pettenkofer

**Decide first. See companion article…**

